# Improved awareness and appropriate use of non-occupational post-exposure prophylaxis (nPEP) for HIV prevention following a multi-modal communication strategy

**DOI:** 10.1186/1471-2458-12-906

**Published:** 2012-10-25

**Authors:** Byron Minas, Sue Laing, Helen Jordan, Donna B Mak

**Affiliations:** 1 Department of Health Western Australia, Communicable Disease Control Directorate, Perth Business Centre, PO Box 8172, Western Australia, WA, 6849, Australia; 2 Centre for Health Policy, Programs and Economics, Melbourne School of Population Health, The University of Melbourne, Victoria, Australia

**Keywords:** Non-occupational post exposure prophylaxis, HIV

## Abstract

**Background:**

In May 2005, the Western Australian Department of Health (WA Health) developed a communication strategy to improve the awareness and appropriate use of non-occupational post-exposure prophylaxis (nPEP) in WA. The communication strategy included the development of an nPEP information pamphlet, the establishment of a 24 hour nPEP phone line and the distribution of the WA Health nPEP guidelines to health professionals. The communication strategy was aimed at gay men, people in sero-discordant relationships, people living with HIV, injecting drug users and health care providers with patients from these populations. This evaluation aimed to assess the awareness and appropriate use of nPEP in WA before and after the commencement of the nPEP communication strategy.

**Methods:**

A program logic method was used to identify the immediate (short-term) and ultimate (long-term) outcomes of the communication strategy. The achievement of these outcomes was evaluated using data from website statistics, a survey of ‘sexuality sensitive’ doctors, statistics published in Perth Gay Community Periodic Surveys (PGCPS) and data from the WA nPEP database. A *χ*^2^ test for trend was conducted to identify any significant changes in the ultimate outcome indicators pre- and post-strategy.

**Results:**

nPEP awareness among gay men in the PGCPS initially increased from 17.2% in 2002 to 54.9% in 2008, then decreased to 39.9% in 2010. After the commencement of the communication strategy, the proportion of nPEP prescriptions meeting the eligibility criteria for nPEP significantly increased (61.2% in 2002-2005 to 90.0% in 2008-2010 (p < .001)). The proportion of nPEP recipients who completed the prescribed course of nPEP (46.6% in 2002-2005 to 66.9% in 2008-2010 (p = .003)) and the proportion who received a post-nPEP HIV test three to four months after the first visit for nPEP (38.8% in 2002-2005 to 51.9% in 2008-2010 (p = .023)) also increased.

**Conclusions:**

Since the introduction of the nPEP communication strategy, the delivery and appropriate use of nPEP have significantly improved in WA. In the 2008-2010 period, an improvement in HIV testing of nPEP recipients at three month follow-up was reported for the first time in WA. However, there is a need for ongoing activities to raise nPEP awareness among gay men.

## Background

Non-occupational post-exposure prophylaxis (nPEP) is a course of antiretroviral drug treatment taken for the prevention of HIV infection after a potential non-occupational exposure to the virus, for example after unprotected sexual contact or the sharing of injecting drug equipment with a person who is HIV positive. The Western Australian Department of Health’s (WA Health) guidelines for the management of nPEP are consistent with the national nPEP guidelines [1] that recommend a four week course of nPEP to be commenced as soon as possible and within 72 hours of a risk exposure for HIV transmission and HIV testing at commencement of nPEP, and at four weeks, three and six months thereafter. Under the guidelines, the risk of HIV transmission is determined by the type of exposure (e.g. receptive anal intercourse, receptive vaginal intercourse) and the risk that the potential source is HIV positive (e.g. a person from a high HIV prevalence country, a man who has sex with other men).

In accordance with the WA nPEP guidelines, WA Health commenced the collection of de-identified information on nPEP recipients in 2002, and preliminary analysis showed that in many cases nPEP was not prescribed in accordance with the recommendations outlined in the WA nPEP guidelines. Results from the Perth Gay Community Periodic Surveys (PGCPS) in 2002 and 2004 also showed low awareness of nPEP availability [2]. In response to these findings WA Health partnered with the WA AIDS Council and specialist sexual health doctors to develop a communication strategy to promote awareness and the appropriate use of nPEP. The strategy, implemented from May 2005 onwards, targeted gay men, people in sero-discordant relationships, people living with HIV, and injecting drug users. The strategy also aimed to raise awareness about the WA nPEP guidelines among health professionals working with these populations.

As part of this strategy, an nPEP information pamphlet and other promotional materials were developed and distributed through the gay press, sexual health services, the WA AIDS Council website and organisations working with the target populations. A free 24 hour phone line staffed by nurses was established to respond to nPEP queries from people who may have had non-occupational exposure to HIV. The WA nPEP guidelines were revised and distributed to relevant health professionals, including doctors authorised to prescribe government funded HIV medication, emergency department staff, and doctors whose practice population includes a significant proportion of gay, bisexual and MSM (men who have sex with men) patients (termed ‘sexuality sensitive’ doctors). These health professionals were also informed about nPEP through professional development sessions, letters and newsletters. In March 2006, WA Health started auditing the WA nPEP database to identify clients who had not been followed-up in accordance with the WA nPEP guidelines; audit results are fed back to staff at nPEP prescribing clinics.

An evaluation of the communication strategy in 2009 reported significant improvements in the prescription of nPEP in accordance with the WA nPEP guidelines in the three year period following commencement of the strategy (May 2005 to April 2008) [3]. However, the previous low levels of follow-up HIV testing of nPEP recipients were unchanged [3]. An increase in nPEP awareness among gay men in Perth was also reported, nearly doubling between 2004 (pre-strategy) and 2006 (post-strategy) [3]. Since these findings, the communication strategy has continued and this evaluation aimed to assess changes in:

• the level of awareness of nPEP among gay men and health professionals,

• the appropriate delivery and use of nPEP in adherence to the WA nPEP guidelines, and

• the use of online nPEP resources

following commencement of the nPEP communication strategy, between 2005 and 2010. The use of online nPEP resources was also evaluated for 2011.

## Methods

**Figure 1:**
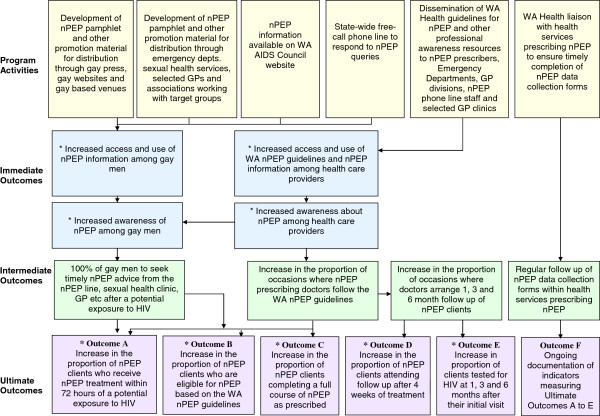
WA nPEP communication strategy: activities and planned outcomes * Outcomes that were assessed in this evaluation.

A program logic [4] of the nPEP communication strategy was developed to identify and select the outcomes for measurement. Immediate (short-term), intermediate (medium-term) and ultimate (long-term) outcomes of each component of the communication strategy are shown in Figure 1. A number of evaluation tools were then developed to measure progress against selected outcomes (Figure 1). It was assumed the evaluation of ultimate outcomes would provide a fair indication of whether the intermediate outcomes had occurred. The data analysed included existing WA Health data, new data collected as part of the evaluation and published PGCPS results described below.

### Immediate outcomes

#### Access to nPEP information

Access to nPEP information was measured by monitoring the number of visits to online nPEP resources. The number of hits for nPEP resources on the WA AIDS Council website (aimed at people at risk of acquiring HIV) was measured during the period November 2008 to April 2011 (data before November 2008 were unavailable). The number of times the WA nPEP guidelines (aimed at health care providers) were downloaded each month from the WA Health website was collected during November 2007 to December 2011. This is the period when the current version of guidelines was available online. The number of unique visitors to these websites was not available.

#### Awareness of nPEP among gay men

Gay men’s awareness of nPEP availability was assessed using published PGCPS results from 2002 to 2010 [2,5-7]. Since 2002 the PGCPS has surveyed respondents biennially on their awareness of nPEP availability. These data were previously analysed using PGCPS results from 2002 (pre-strategy), 2004 (pre-strategy) and 2006 (post-strategy) [3]. The assessment was extended to PGCPS results from 2008 and 2010 in this evaluation. The PGCPS is a cross-sectional survey of sexual behaviour, testing for HIV and other sexually transmissible infections, and drug use among gay-community-attached men in Perth, and is part of a larger national survey conducted in other Australia cities. Further details about the PGCPS are available online [7].

#### Awareness of nPEP among health care providers

In May 2011, ‘sexuality sensitive’ doctors recognised by the WA AIDS Council were invited to complete an online survey to assess their level of awareness about the availability of nPEP and the WA nPEP guidelines. Doctors who had not completed the survey after one month were sent a reminder offering them one additional week for completion.

### Ultimate outcomes

#### nPEP treatment practices and follow-up testing

De-identified data on all clients prescribed nPEP in WA are collected by the prescribing doctor and recorded on the WA nPEP database with the client’s informed consent. The data include client demographics, risk exposure details, HIV test results and whether clients completed the prescribed treatment.

These data were analysed to determine if there were any significant changes among nPEP clients in relation to the following indicators:

• Commencement of nPEP within 72 hours of a risk exposure to HIV

• Eligibility for nPEP according to WA nPEP guidelines

• Level of completion of prescribed nPEP treatment

• Testing for HIV at the initial visit and follow-up testing in accordance with the WA nPEP guidelines

• The number and proportion of positive HIV test results.

While the WA nPEP guidelines recommend follow-up HIV testing at four weeks, three months and six months after the initial visit, this evaluation examined the number and proportion of nPEP clients who were tested at four to six weeks, three to four months and six to seven months follow-up, in recognition of clinicians’ efforts to recall clients following a non-attendance.

Data were categorised into three different reporting periods for analysis: nPEP recipients who had a risk exposure between May 2002 and April 2005 (Reporting Period One), May 2005 and April 2008 (Reporting Period Two), and May 2008 and December 2010 (Reporting Period Three). A *χ*^2^ test for trend was conducted to identify any significant change over time for the three reporting periods in relation to the aforementioned indicators. The data were analysed using Microsoft Access 2003, Microsoft Excel 2003 and IBM SPSS Statistics 19.

Ethics approval was not required because the data were collected as part of routine quality improvement of an existing disease control program.

## Results

### Immediate outcomes

#### Awareness of nPEP among gay men

**Figure 2:**
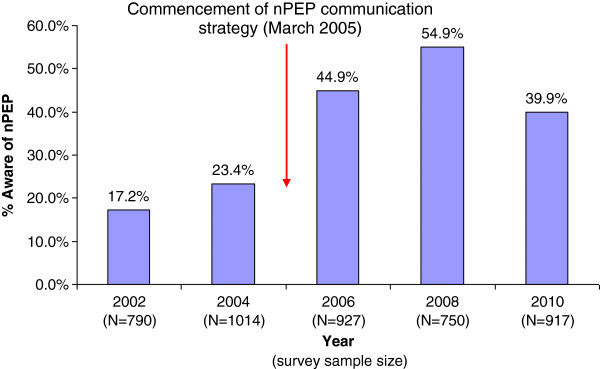
**Proportion (%) of gay men aware that nPEP was currently available (Perth Gay Community Periodic Surveys 2002 to 2010[**[2]**,**[5-7]**]).**

The proportion of PGCPS respondents aware that nPEP was available increased from 2002 (17.2%) to 2008 (54.9%). The increase was most notable between the 2004 and 2006 surveys. However, awareness decreased to 39.9% by 2010 (Figure 2).

#### Awareness of nPEP among health care providers

The nPEP awareness survey of ‘sexuality sensitive’ doctors was completed by 27.8% of the 36 doctors invited to participate. Seven respondents were aware nPEP was available and five were aware of the WA nPEP guidelines. Professional development events (n = 3), the nPEP pamphlet (n = 3) and colleagues (n = 3) were the most common sources of information on nPEP.

#### Visitors to online nPEP resources

From November 2007 to December 2011, the WA nPEP guidelines were downloaded 801 times (176 in 2008, 204 in 2010 and 198 in 2011) from the WA Health website (average 16 times/month, range 10-29 downloads/month). Between November 2008 and December 2011, the nPEP resources posted on the WA AIDS Council website received 2293 hits.

### Ultimate outcomes

#### nPEP treatment practices and follow-up testing

During Reporting Period One, 48.6% of nPEP clients were women who were prescribed nPEP after a sexual assault. The majority of these women (68.0%) did not meet the WA nPEP guidelines for eligibility as their assailant was not known to be HIV positive or not considered at risk of HIV infection, e.g. a person from a high HIV prevalence country. Since the communication strategy has been in place (Reporting Periods Two and Three), the number of males who reported high risk sexual contact with another male significantly increased as a proportion of all nPEP recipients (Table 1), and females prescribed nPEP after a sexual assault significantly decreased to 13.1% (p < 0.001) of all nPEP recipients. As a result, an overall increase was seen in the proportion of nPEP prescriptions meeting the eligibility criteria based on the nPEP guidelines (Table 1).

**Table 1:** Indicators of adherence to WA nPEP guidelines - time of commencement, nPEP eligibility and HIV testing

	**Reporting period one**	**Reporting period two**	**Reporting period three**	***χ***^**2**^**test for trend**
	**May 2002 to Apr 2005 n (%)**	**May 2005 to Apr 2008 n (%)**	**May 2008 to Dec 2010 n (%)**	**(p-value)**
**Total number of nPEP recipients**	103	130	160	
**nPEP treatment commenced within 72 hours of potential exposure to HIV**	82 (79.6)	111 (85.4)	136 (85.0)	NS
**Eligible for nPEP based on WA nPEP Guidelines**	63 (61.2)	115 (88.5)	144 (90.0)	p < .001
**Risk exposure to HIV**				
High risk male sexual contact with HIV positive male	7 (6.8)	17 (13.1)	31 (19.4)	p = .004
High risk male sexual contact with male of unknown HIV status	23 (22.3)	64 (49.2)	68 (42.5)	p = .004
High risk heterosexual contact with HIV positive person	8 (7.8)	14 (10.8)	13 (8.1)	NS
High risk heterosexual contact with person from HIV risk population (e.g. person from high HIV prevalence country)	21 (20.4)	18 (13.8)	28 (17.5)	NS
Other high risk exposure	4 (3.9)	2 (1.5)	4 (2.5)	NS
**Completed nPEP treatment as prescribed**	48 (46.6)	70 (53.8)	107 (66.9)	p = .003
**HIV Test**				
At initial visit	94 (91.3)	126 (96.9)	148 (92.5)	NS
4 to 6 weeks after initial visit	48 (46.6)	71 (54.6)	94 (58.8)	NS
3 to 4 months after initial visit	40 (38.8)	49 (37.7)	83 (51.9)	p = .023
6 to 7 months after initial visit	20 (19.4)	34 (26.2)	44 (27.5)	NS

*NS* = Not significant.

The majority of nPEP recipients in each Reporting Period were tested for HIV at their initial visit for nPEP and commenced treatment within 72 hours of their risk exposure (Table 1). The proportion of nPEP recipients who completed their prescribed course of treatment significantly improved between Reporting Periods One and Three (Table 1).

There was a small, non-significant, improvement in the proportion of clients tested for HIV at four to six weeks after the initial visit, a greater and statistically significant increase at three to four months (most evident in Reporting Period Three), and no improvement at six to seven months (Table 1). The reasons for non-testing were not documented systematically. However, comments recorded as free text indicated that many clients did not return for testing as they had relocated interstate or overseas, or had been offered but declined follow-up testing.

Between May 2002 and August 2011, two nPEP recipients tested positive for HIV at six to seven month follow-up. Both clients were HIV negative when they commenced nPEP and one tested negative for HIV three months thereafter. However it was difficult to conclude whether infection was due to the reported exposure as both had risk exposures to HIV post nPEP.

## Discussion

Since the nPEP communication strategy was established, inappropriate nPEP use for low risk exposures has decreased, and completion of nPEP treatment and HIV testing of nPEP clients at three to four month follow-up have increased. Between 2008 and 2010, nPEP awareness among gay men participating in the PGCPS decreased after having increased since 2002.

Similar decreases were also reported from Gay Community Periodic Surveys in other states [8,9]. However in these states, at least 50% of participants in 2010 knew that nPEP was currently available, a higher proportion than that seen in Perth (40%). In previous nPEP awareness surveys in the US [10] and the UK [11], awareness ranged from 36% [10] to 56% [11] among MSM.

The 2010 PGCPS had a significantly higher proportion of participants under the age of 25 years (40.0%) compared to the previous surveys (21.9% to 27.6%), which may have accounted for the apparent decline in nPEP awareness among PGCPS respondents. Another possible reason for this decline could lie in the methods used as part of the communication strategy. The promotion materials used to raise awareness about nPEP, and the channels used to disseminate these materials, have only changed slightly since the communication strategy began in 2005. The PGCPS results from 2010 suggest that a fresh approach should be considered, which could include opportunities to engage recent trends in social marketing, such as the use of social media.

The improvement in nPEP prescribing practices and rate of completion previously reported in WA [3] were sustained in the May 2008 to December 2010 period (Reporting Period Three). The nPEP completion rate in WA is similar to the rate reported in an nPEP study in the UK [12].

In contrast to the evaluation of the nPEP communication strategy in 2009 [3], an improvement in testing rates among nPEP recipients was observed for the first time in WA. The rate of post-nPEP HIV testing three to four months after the initial visit increased from 38% to 51.9% between Reporting Periods Two and Three. This compares favourably with the Victorian nPEP program which reported that 34% of nPEP clients had been tested at three month follow-up [13]. Hospitals in the UK [14] and France [15] have also reported low follow-up testing of nPEP clients after three months (35% and 29% respectively). Studies examining testing rates over time further highlight the challenge of improving post-nPEP testing. For example a UK study of nPEP recipients found no significant change in HIV testing rates at three month follow-up, before and after the introduction of the national nPEP guidelines and a communication strategy [12]. The higher rate of follow-up testing in WA may reflect inclusion of clients tested at the four month period in the analysis.

The recommendation for HIV testing beyond the three month follow-up period will soon be removed from the revised national nPEP guidelines. This is consistent with the British Association for Sexual Health and HIV (BASHH) guidelines in the UK [16]. With only a minority of clients tested after six months, the removal of six month HIV testing from the WA nPEP guidelines would enable clinicians to focus efforts on recalling patients to be tested at three to four months, particularly as recent progress has been seen for this period of follow-up.

A number of limitations need to be considered in the interpretation of these evaluation findings. Firstly, while the majority of 'sexuality sensitive' doctors surveyed in this evaluation were aware about the availability of nPEP, this was limited by the low survey response rate. Secondly, the evaluation of nPEP awareness among health care providers focused solely on ‘sexuality sensitive’ doctors. The generalisation of these evaluation findings as an indicator of nPEP awareness among ‘sexuality sensitive’ doctors and other relevant health care providers is therefore limited. Finally, it is difficult to conclude from the methods used in this evaluation the extent to which the communication strategy has led to the improvements reported here. This evaluation was a pre- and post-study of the nPEP communication strategy and potential confounders which may have contributed to the observed results were not examined. Exploring the views of nPEP clinicians and clients in future evaluations could identify whether the communication strategy has influenced nPEP awareness and practices.

## Conclusions

The decrease in nPEP awareness seen among PGCPS respondents signals the need for ongoing activities to raise awareness among WA gay men and other MSM, particularly those younger than 25 years. An additional question could be included on the nPEP data collection form and/or the PGCPS questionnaire to determine whether people became aware of nPEP through activities implemented as part of the communication strategy.

Apart from this evaluation few other studies have monitored HIV testing of nPEP clients over time and observed an improvement in post-nPEP testing rates. Future studies on long-term trends, particularly after a campaign or initiative to improve testing rates, could help identify strategies to improve follow-up testing.

## Abbreviations

BASHH: British Association for Sexual Health and HIV; MSM: Men who have sex with men; nPEP: Non-occupational post-exposure prophylaxis; PGCPS: Perth Gay Community Periodic Survey; WA: Western Australia; WA Health: Western Australian Department of Health.

## Competing interests

The authors declare that they have no competing interests.

## Authors' contributions

BM participated in the design of the study, data collection and conducted statistical analysis. SL participated in the design of the study and data collection. DM participated in the design of the study and supervised the study. HJ participated in the design of the study and provided advice on evaluation methods including the program logic. All authors contributed to drafts of the manuscript, and have read and approved the final manuscript.

## Authors' information

BM maintains the WA nPEP Database at the Department of Health Western Australia. This includes the entry, cleaning and analysis of data on the database. HJ is a lecturer and researcher at the University of Melbourne with expertise in health program evaluation, epidemiology and health policy.

## Pre-publication history

The pre-publication history for this paper can be accessed here:

http://www.biomedcentral.com/1471-2458/12/906/prepub
